# Rights of Authors

**Published:** 1858-04

**Authors:** 


### Rights of Authors.

We have received from Prof. Martin Paine, of New York, a portion of the Institutes of Medicine, embracing a chapter upon the "Rights of Authors," the object of which is to set forth the principal details of what he considers himself the unquestionable author in reference to the recent conflicting claims as to priority in certain physiological discoveries. He remarks:

"Upon all questions of priority that concern the advancement of Science and Art, there is, doubtless, a general understanding that the principle should not only be sacredly observed, but that, whenever violated, there should be a common effort to repair the injury. This is alike due to the individual, to the principle, and to the common good. Nor is it less the privilege of the individual, who may have good reason to think that the principle has not been extended to himself, to vindicate his rights, and to appeal to that sympathy which forms the bond of union among honorable men. It is a common cause, and not seldom demands protection.

"The writer is very sensible that unaccountable coincidences often present themselves in the development of new thoughts, and in the discovery of hidden things, especially where enduring reputation may be won. "*Ubi mel, ibi apes.*"—"*Uno tiene la fama, y otro carda la lana.*" But the reader, with these Institutes before him, will quickly find that much that is claimed by Dr. Hall, and all that he has granted to Dr. Campbell, in the foregoing quotation, therefore, all that Dr. Allen appropriates to himself,* abounds in this volume, and, in fact, constitutes the life and soul of the work, as it does, also, of the "*Commentaries*," and of the Essay on the "*Modus Operandi of Remedies*;" nor can the reader fail of the conclusion that, were Dr. Hall's "adjudication," and Dr. Allen's after-thought, founded in any justice, and were not the claimants themselves the obnoxious parties, the present writer would have been long ago convicted by them and by others of arrogant assurance and the grossest plagiarisms. Nevertheless, the Author is most happy to find that his solitary position is becoming relieved, and that a practical direction has been given to his labours by others which cannot fail of carrying forward the great doctrines at which he has toiled, and against manifold obstacles during his professional life."

---

* "Unus utrique
Error; sed variis illudit partibus."—HORACE

